# Adherence to the Mediterranean Diet and Its Association with Body Composition and Physical Fitness in Spanish University Students

**DOI:** 10.3390/nu11112830

**Published:** 2019-11-19

**Authors:** Ana Isabel Cobo-Cuenca, Miriam Garrido-Miguel, Alba Soriano-Cano, Asunción Ferri-Morales, Vicente Martínez-Vizcaíno, Noelia María Martín-Espinosa

**Affiliations:** 1 Grupo Interdisciplinar en Cuidados IMCU, Universidad de Castilla la Mancha, 45071 Toledo, Spain; Anaisabel.cobo@uclm.es; 2 Health and Social Research Center, Universidad de Castilla-La Mancha, 16071 Cuenca, Spain; Alba.soriano@uclm.es (A.S.-C.); Asuncion.Ferri@uclm.es (A.F.-M.); vicente.martinez@uclm.es (V.M.-V.); 3 Facultad de Ciencias de la Salud, Universidad Autónoma de Chile, 1101 Talca, Chile; 4 Facultad de Fisioterapia y Enfermería, Universidad de Castilla la Mancha, 45071 Toledo, Spain; Noelia.martin@uclm.es

**Keywords:** Mediterranean diet, physical fitness, cardiovascular fitness, body composition

## Abstract

The aims of this study were to assess the association of adherence to the Mediterranean diet (MD) with physical fitness and body composition in Spanish university students and to determine the ability to predict the MD adherence of each Mediterranean Diet Adherence Screener (MEDAS) item. A cross-sectional study was performed involving 310 first-year university students. Adherence to the MD was evaluated with MEDAS-14 items. Anthropometric variables, body composition, and physical fitness were assessed. Muscle strength was determined based on handgrip strength and the standing long jump test. Cardiorespiratory fitness (CRF) was measured using the Course–Navette test. Only 24% of the university students had good adherence to the MD. The ANCOVA models showed a significant difference between participants with high adherence to the MD and those with medium and low adherence in CRF (*p* = 0.017) and dynamometry (*p* = 0.005). Logistic binary regression showed that consuming >2 vegetables/day (OR = 20.1; CI: 10.1–30.1; *p* < 0.001), using olive oil (OR = 10.6; CI: 1.4–19.8; *p* = 0.021), consuming <3 commercial sweets/week (OR = 10.1; IC: 5.1–19.7; *p* < 0.001), and consuming ≥3 fruits/day (OR = 8.8; CI: 4.9–15.7; *p* < 0.001) were the items most associated with high adherence to the MD. In conclusion, a high level of adherence to the MD is associated with high-level muscular fitness and CRF in Spanish university students.

## 1. Introduction

The influence of diet on human health has aroused the interest of epidemiologists for hundreds of years. Among different dietary patterns, the Mediterranean diet (MD) has become very popular over the last decades, since the Seven Countries study reported the benefits of this diet for the prevention of a range of chronic disease outcomes [1]. Nowadays, some population-based studies and randomized trials have provided evidence suggesting that greater adherence to the MD reduces the risk of cardiovascular diseases, cancer, neurodegenerative diseases, diabetes, and all-cause mortality [2,3,4,5]. A meta-analysis demonstrated that a two-point increase in an adherence score was associated with a significant reduction in overall mortality and a reduced risk of cardiovascular diseases, cancer, and neurodegenerative diseases [6]. The MD represents a dietary pattern that incorporates healthy traditional eating habits of populations from countries surrounding the Mediterranean sea which includes high consumption of vegetables, fruit, legumes, nuts, beans, whole grains, grains, fish, and unsaturated fats, such as olive oil, and low consumption of red meat and dairy products, although the relationship between dairy consumption and health has been under discussion in recent years [7,8].

In the last fifteen years, several validated scores have been used to measure the adherence to the MD; although their reliability has been proven, more research is needed to determine their quality and cultural adaptation [9]. The Mediterranean Diet Adherence Screener (MEDAS-14 items) is a valid instrument for the estimation of adherence to the MD which has demonstrated itself to be sensitive to capturing the inverse association between MD and obesity indicators in high cardiovascular risk adults; moreover, this 14-item questionnaire requires less involvement from participants than the usual food frequency questionnaires and is less time demanding [10].

Physical fitness is robustly associated with a decrease in cardiovascular mortality as well as the risk of developing cardiovascular disease [11,12,13]. Recently, some studies have confirmed that higher levels of muscular strength are associated with a reduced risk of all-cause mortality in adults [14,15], and studies have also shown the beneficial effect of higher muscular strength in both children and adolescents [16,17].

The transition to college or university is a critical period for young adults, who are often facing their first opportunity to make their own food decisions [18,19]. Sometimes, the lack of time and monetary concerns may determine the adoption of unhealthy eating habits and sedentary patterns [20,21], leading to increased weight [22,23] which may persist during adulthood.

Although MD adherence has been studied in relation to physical fitness and body composition in different stages of life [24,25], research focused on the young adult group is scarce [26]. In this sense, the objectives of this study were (1) to estimate the prevalence of the adherence to the MD, (2) to assess the association of adherence to the MD with physical fitness and body composition in a sample of Spanish university students during their first year at university, and (3) to know the ability of each MEDAS item in the same sample to predict MD adherence.

## 2. Materials and Methods 

### 2.1. Study Design and Participants

A cross-sectional observational study was performed with first-year university students, aged 18–30 years, during the 2017–2018 academic year belonging to the Faculties of Education, Nursing, Physiotherapy, and Social Work and Polytechnic, from the Albacete, Cuenca, and Toledo campuses of the University of Castilla-La Mancha, Spain. A total of 560 students were invited, and 310 (55.33%) agreed to participate. Of these 310 participants, only 239 university students’ total lean mass and total fat mass (by dual energy X-ray absorptiometry (DXA)) were measured. The participants included in this subsample did not differ in age, sex, or parental socioeconomic status from the whole sample of young adults participating in the study.

This report is part of the study: “Lifestyle, adiposity and vascular function in university students from Castilla-La Mancha, Spain”. The Clinical Research Ethics Committee of the “Virgen de la Luz” of Cuenca approved the study, and the investigation adhered to the principles of the Declaration of Helsinki (REG: 2016jPI1116). Researchers explained the purpose of the study to eligible students: participating university students should not have any type of physical or mental disorder that prevented them from participating in physical measurements or completing questionnaires about lifestyle habits. Those who decided to participate in the study read and signed the informed consent form.

### 2.2. Sample Size

For a 95% confidence level, the minimal sample size, calculated using Epidat 4.2, was 300 students considering an obesity prevalence of 23% [27], a precision of 5%, and a 20% non-response rate. Taking as a sample frame the list of enrolments of these careers, a random 560 students were invited, from which 310 students agreed to participate.

### 2.3. Adherence to the Mediterranean Diet

All participants completed two self-reported questionnaires: (a) The Mediterranean Diet Adherence Screener (MEDAS) [28], a Spanish-validated questionnaire that consisted of 14 items that included 12 questions about the frequency of food intake (olive oil, vegetables, fruit, red meat, animal fats, carbonated beverages, red wine, fish/seafood, nuts, commercial food, traditional dishes (with tomato sauce, garlic, onion, etc.) and two questions about the preferred cooking fat used and meat consumed. Each item was scored as zero or one, and the final score is the sum of each (0–14). (b) The Food-Frequency Questionnaire (FFQ) was used to determine the total intake of energy, carbohydrates, proteins, and fats [29]. The FFQ is a validated questionnaire with 137 items scored on a Likert scale with nine levels of frequency of consumption (never or almost never, 1–3 times per month, once per week, 2–4 times per week, 5–6 times per week, once per day, 2–3 times per day, 4–6 times per day, and more than six times per day). 

### 2.4. Anthropometrics and Body Composition Measures

The students wore light clothes and were barefoot. Weight, height, and waist circumference were evaluated twice at a 5 min interval. Weight (kg) was obtained using a SECA-7 scale. Height was measured using a wall-mounted stadiometer (Seca222, Vogel and Halke) with participants barefoot and standing against the wall with their chin parallel to the floor. Body mass index (BMI) (weight (kg)/height (m^2^)) was categorized into three groups: normal weight (18.5 ≤ BMI ≤ 24.9), overweight (25 ≤ BMI ≤ 29.9), and obesity (BMI ≥ 30) [30]. Waist circumference (cm) was measured at the end of exhalation in the middle point between the costal margin and iliac crest. Body fat percentage (BF%), total fat mass (kg), and total lean mass (kg) were obtained using dual-energy X-ray absorptiometry (DEXA) (Lunar iDXA, GE Medical Systems Lunar, Madison, WI 53718, USA). The students were scanned in a supine decubital position. All scans were performed at high resolution by two trained researchers. 

### 2.5. Sociodemographic and Lifestyle Variables

For each enrolled student, a self-reported questionnaire including the following items was used: (1) Type of housing: family dwelling; university residence or shared apartment. (2) Lifestyle smoking habit, categorized as: non-smoker, who has not smoked more than 100 cigarettes in their lifetime and does not currently smoke; ex-smoker, who has smoked more than 100 cigarettes in their lifetime but has not smoked in the last 28 days; smoker, who smokes regularly. (3) Lifestyle alcohol habits: Never, 1–4 times a month, and ≥2 times a week.

### 2.6. Physical Fitness

#### 2.6.1. Muscle Strength 

Handgrip strength was evaluated using a dynamometer (TKK 5401 Grip-D; Takey, Tokyo, Japan) and was adjusted for the sex and hand size of each participant. Lower limb strength was measured using the standing long jump test; students jumped from a starting line with both feet aligned. Both tests were performed twice, and the data from the best trial were recorded. With the data of the two strength tests, a strength index that consisted of the sum of the standardized z-scores of handgrip/weight and standing long jump was calculated. The best score recorded was used for analysis.

#### 2.6.2. Cardiorespiratory Fitness (CRF)

Cardiorespiratory fitness (CRF) was measured using the Course Navette test (20 m shuttle run test (20 m SRT)). Participants had to run between two lines separated by a distance of 20 m, while keeping the rhythm of audio signals emitted by a pre-recorded compact disc. The initial speed was 8.5 km/h, which increased by 0.5 km/h every minute. Participants were encouraged to continue running as long as possible during the course of the test. We recorded the last half stage completed as an indicator of their CRF. Leger’s formula was used to obtain estimations of submaximal oxygen consumption (VO2) (31.025 + (3.238 × velocity) − (3.248 × age) + (0.1536 × age × velocity)) [31].

#### 2.6.3. Physical Activity

The total amount of physical activity was objectively measured using GENEActive accelerometers (ActivInsights). Participants wore these devices set at a fixed frequency of 30.0 Hz on their wrists for 7 consecutive days (including nights) for collecting raw acceleration data measured in “g” for each movement axis (x, y, and z), in order to estimate the participants’ physical activity. We considered as valid measurements those of ≥5 days, including 1 weekend day. For this study, mean total minutes/day of physical activity was estimated [32].

### 2.7. Statistical Analysis

For a descriptive analysis of the quantitative variables, the mean (m) and standard deviation (SD) were calculated; for categorical variables, count (*n*) and percentages (%) were used. We also compared proportions of categorical variables using chi-squared tests for contingency tables and student’s *t*-test for quantitative variables. 

The MEDAS scores were categorized as low adherence (<9) and good adherence to the Mediterranean dietary pattern. Since recent studies have suggested that the relationship between adherence to the MD and cardiometabolic risk might have a linear relationship, we also categorized the adherence to the MD into three levels of adherence (low, first quartile; medium, second and third quartiles; and high, fourth quartile). To test mean differences in energy intake, body composition and fitness parameters by categories of MEDAS, ANCOVA models were calculated. Sex, age, smoking habits, alcohol habits, and type of housing were included as covariates in Model 1. Pairwise post-hoc comparisons were examined using the Bonferroni test

To quantify the predictive ability of each MEDAS item for adherence to the Mediterranean dietary pattern, logistic binary regression models were estimated using adherence to MD (low/good) as a dependent variable and each of the items of adherence to the MD questionnaire as independent variables. Statistical significance was set at *p* < 0.05. We used the program IBM SPSS Statistics version 24 (IBM Corp., Armonk, NY, USA).

## 3. Results

The sample consisted of 310 students, 64.5% (202) of whom were women. The average age was 20.9 (SD 2.5) years. Weight, height, and total lean mass were significantly higher in men than in women (*p* < 0.001). The mean BF%, total fat mass, and BMI were higher in women than in men (*p* < 0.001). A total of 72.8% of the participants were categorized as normal weight and 21.4% were categorized as overweight (26.2% in men, 19.2% in women). Men showed higher muscular fitness z-scores and aerobic capacity (VO_2_ max) than women (Table 1).

Table 2 shows the agreement with the dietary recommendations based on 14 items from the FFQ and cooking preferences (questionnaire of Mediterranean diet adherence) by sex [28]. A very low adherence was observed in each of the items of the MEDAS questionnaire (14 items). 

Table 3 (model 0) shows that participants classified as having high adherence to the Mediterranean dietary pattern showed higher weight, BMI, and total lean mass (kg) than participants with medium and low adherence (*p* < 0.05). In addition, they also showed significantly higher CRF, z-scores of muscular strength index, handgrip strength (kg), and standing long jump (cm) than participants with medium and low adherence. Finally, the dietary pattern of those with higher adherence to the MD contained a higher percentage of protein and a lower percentage of fat than those with medium and low adherence (*p* < 0.05). However, the differences with BMI, total lean mass, and standing long jump did not reach statistical significance when controlling for potential confounders (model 1).

Table 4 shows, in a logistic binary regression model, that 12 of the 14 items of the MEDAS score were associated with a high adherence to the Mediterranean dietary pattern. To consume >2 servings of vegetables per day (OR = 20.1; CI: 10.1–30.1; *p* < 0.001), to use olive oil (OR = 10.6; CI: 1.4–19.8; *p* = 0.021), to consume <3 commercial sweets or pastries per week (OR = 10.1; IC: 5.1–19.7; *p* < 0.001), and to consume three or more fruits per day (OR = 8.8; CI: 4.9–15.7; *p* < 0.001) were the items more closely associated with high MD adherence. Only the following two items were not significantly associated with the Mediterranean dietary pattern: “How many servings of butter, margarine, or cream do you consume per day?” and “How many sweet or carbonated beverages do you drink per day?”.

## 4. Discussion

In this study, we evaluated the prevalence of the adherence to the MD and the relationship between adherence to the MD and physical fitness and body composition in a sample of Spanish university students during their first academic year. The main findings of this study were as follows: (1) the low prevalence of good adherence to the Mediterranean dietary pattern; (2) in those with good adherence to MD, the daily protein intake was higher than in those with medium–low adherence, while the fat intake was lower; (3) the good adherence group had significantly higher levels of physical fitness (CRF and muscular fitness); and (4) 12 of the 14 items of the MEDAS score were associated with a high adherence to the Mediterranean dietary pattern.

A great variability in the dietary patterns of university students has been reported [33,34], even in studies conducted in the same country. In Mediterranean countries (Greece, Italy, Spain), adherence to the MD ranges from 20–30% among students with lower prevalence rates [35,36,37,38] to more than 40% [39] or more than 70% [40] in those with higher prevalence rates. In our study, only 24% of the students scored as having good adherence to the MD. This variability could be because different questionnaires have been used to evaluate adherence to the MD. In a systematic review of the instruments for quantifying MD adherence, Zaragoza et al. [9] concluded that the psychometric properties and applicability parameters of most scores had not been tested; thus, only studies using the same tools could provide a good picture of the variability in the prevalence of MD adherence. Apart from this variability, in general, studies report low prevalence rates of adherence to the MD because young people tend to abandon the Mediterranean dietary pattern [41,42,43,44] and, in general, to move away from healthy lifestyle habits [45]. In a study on preferences when choosing food, students considered taste and pleasure as the main factors influencing their choice and that they did not take into account the importance of eating healthy food [46]. In addition, social pressure, the high cost of healthy food [41,46], and short time for eating were decisive factors regarding the non-adherence to healthy diets [46]. 

In our sample, students with good adherence to the MD ingested more calories and protein and less fat than those with medium or low adherence. Similar results were found in other studies [9,37,43]. Furthermore, considering the food groups, the consumption of vegetables, olive oil, and fruits was positively associated with MD adherence, as has been described in other research [9,43,47]. The consumption of these foods is poor in the university population (possibly because of the price, lack of time, taste, etc.) both in Mediterranean and non-Mediterranean countries [33,34]. 

Although adherence to the MD diet was only acceptable, in our sample, more than 90% of participants used olive oil as a culinary fat, and less than 5% used another fat (margarine, butter, etc.). In Spain, the consumption of olive oil is very common, and olive oil is not expensive [40]. The use of oil olive as the main fat is attributable to the cultural heritage of Mediterranean countries. Olive oil reduces the incidence of cardiovascular diseases and increases HDL cholesterol, among other effects [48,49].

The influence of MD adherence on some anthropometric parameters is a controversial issue. Thus, while some studies suggest that adherence to this dietary pattern is associated with lower adiposity [50], others have reported that MD adherence did not predict weight status [51,52] or changes in WC [53]. In our sample, adherence to the MD was not associated with fat mass or WC, although those with higher adherence to the MD had higher levels of BMI and total lean. This fact may be explained by the fact that the MD is not a low caloric diet because of its high content of mono- and polyunsaturated fats and carbohydrates, all of which are potentially responsible for this weight increase [54]. In our study, at least part of this increase in lean mass may be attributable to practising more physical activity in the higher adherence to MD group. In addition, consistent evidence supports that muscle mass plays an important role in the prevention of some chronic diseases such as obesity and diabetes [55,56].

Recent research conducted in adolescents and young adults supports the importance of muscular fitness in cardiovascular disease prevention. In a sample of 1248 college students, Ramirez-Velez et al. [26] found that those with optimal MD adherence and muscular fitness showed the healthiest cardiometabolic profile. Similar results were reported in another study including 2444 adolescents in which two components of physical fitness, aerobic capacity and muscular fitness, were examined [24]. In line with these findings and those of other studies [57,58,59], our data show that students with high MD adherence presented the highest levels of both cardiorespiratory and muscular fitness. If these results were confirmed in long-term follow-up studies, it would be very important, because the crucial role of physical fitness in reducing morbidity and mortality risk is not a debatable topic [16,58,59,60,61,62,63,64,65]. 

In our study, the analysis of the association of each individual item with the total MEDAS score shows that two items were not associated with the summative score: “How many servings of butter, margarine, or cream do you consume per day?” and “How many sweet or carbonated beverages do you drink per day?”. In the Spanish context, the consumption of both food groups is not as common as in other countries; this may be the reason why these two items did not correlate with MD. According to the “Food Consumption in Spain 2018” report from the Spanish Ministry of Agriculture, Fisheries and Food [66], the aim of which is to determine the type of food consumed by the Spanish population, olive and sunflower oils are used for cooking in 64.9% and 32.1% of Spanish families, respectively, and margarine, butter, or cream represent only 1% of the total dairy intake. Likewise, the consumption of sweet or carbonated beverages in Spain is moderate and has displayed a declining trend during the last years, in such a way that in 2018 alone, Spanish people had reduced their consumption by 4% with respect to the previous year [66]. For this reason, although MEDAS is a validated questionnaire to determine the adherence to the MD, we believe it may be necessary to readjust this questionnaire to fit Spanish people’s dietary patterns [9].

The strengths of this study include the novelty of combined associations of muscular fitness, CRF, body composition, and energy intake with MD in a large sample of college students and the ability to predict the MD adherence of each MEDAS item in the same sample. 

There were some limitations of this study that should be acknowledged. First, its cross-sectional design prevents us from establishing cause–effect inferences. Second, the sample studied included only university students; thus, caution is necessary when making inferences about other age ranges. Third, of the 310 participants in our study, only in 239 university students’ total lean mass and total fat mass (by dual energy X-ray absorptiometry (DXA)) were measured. Fourth, the use of self-reported dietary data could result in underreporting or unintentional measurement errors. In addition, the use of the FFQ-137 [29] and the MEDAS questionnaires [28] have only been validated in the elderly population; thus, the results of this study should be cautiously interpreted due to the potential bias that involves assuming that their validity and reliability could be extrapolated to a different age group in which these properties have never been proven.

## 5. Conclusions

Our results could be important from a public health point of view, since they reveal the low adherence of Spanish university students to the Mediterranean dietary patterns, and this could have long-term health consequences. Reinforcing the importance of Mediterranean dietary patterns for the health of young adults, our data support the association between MD adherence and both cardiorespiratory and muscular fitness. Finally, our data suggest that a MEDAS calibration that considers current Spanish dietary patterns could be suitable. 

## Figures and Tables

**Table 1:** Descriptive characteristics of the study sample by sex.

	All (*n* = 310)	Men (*n* = 108)	Women (*n* = 202)	*p* *
Age (years)	20.9 (SD 2.5)	21.1 (SD 2.8)	20.7 (SD 2.3)	0.146
Weight (Kg)	65.4 (SD 12.3)	72.6 (SD 10.99)	61.4 (SD 11.1)	**<0.001**
Height (cm)	167.3 (SD 8.6)	175.3 (SD 7.0)	162.9 (SD 5.8)	**<0.001**
Waist circumference (cm)	78.9 (SD 9.3)	83.0 (SD 7.9)	76.6 (SD 9.2)	**<0.001**
% Fat mass ^a^	29.3 (SD 9.0)	20.5 (SD 6.3)	33.7 (SD 6.7)	**<0.001**
Total fat mass (Kg) ^a^	18.7 (SD 7.9)	14.4 (SD 5.9)	20.8 (SD 7.9)	**<0.001**
Total lean mass (Kg) ^a^	43.0 (SD 9.3)	53.5 (SD 6.831)	37.8 (SD 4.9)	**<0.001**
BMI (Kg/m^2^)	23.3 (SD 3.5)	23.5 (SD 3.03)	23.1 (SD 3.8)	0.269
Normal weight (%)	72.8	71.4	75.1	0.140
Overweight (%)	21.4	26.2	19.2	
Obesity (%)	4.4	2.4	5.7	
20 m SRT (stages)	5.8 (SD 2.6)	7.0 (SD 1.9)	3.8 (SD 1.4)	**<0.001**
CRF (VO_2_ max estimate)	33.4 (SD 9.9)	40.4 (SD 8.9)	28.7 (SD 7.6)	**<0.001**
Muscle strength (cm/Kg) ^b^	0.013 (SD 1.7)	1.523 (SD 1.2)	−1.050 (SD 1.2)	**<0.001**
Standing long jump (cm)	161.2 (SD 43.7)	195.4 (SD 31.9)	136.8 (SD 33.5)	**<0.001**
Dynamometry (kg)	30.4 (SD 9.5)	39.2 (SD 7.7)	24.4 (SD 4.7)	**<0.001**
Physical activity (min/day)	223.1 (SD 65.3)	221.4 (SD 76.9)	223.6 (SD 67.6)	0.880
EI (Kcal)	2795.7 (SD 1804.7)	2865.9 (SD 1287.0)	2757.6 (SD 2033.2)	0.590
Carbohydrate (% EI)	43.0 (SD 7.1)	43.1 (SD 6.6)	42.9 (SD 7.3)	0.852
Protein (% EI)	17.4 (SD 3.4)	17.4 (SD 3.2)	17.5 (SD 3.6)	0.749
Fat (% EI)	38.2 (SD 6.2)	37.9 (SD 5.9)	38.3 (SD 6.3)	0.578
Lifestyle smoking habits (%)				
Non-smoker	81.3	82	80.9	0.580
Ex-smoker	4.5	5.7	3.9	
Smoker	14.2	12.3	15.2	
Lifestyle alcohol habits (%)				0.173
Never	10.1	12.7	8.6	
1–4 times a month	88.2	84.1	90.6	
≥2 times a week	1.7	3.2	0.9	
Type of housing (%)				**0.019**
Family dwelling	41.3	50.4	36.4	
University residence	19.8	19.7	19.9	
Shared apartment	38.8	29.9	43.7	
Adherence Mediterranean diet (%)				0.090
Low adherence	65.4	70.4	79.0	
Good adherence	24.0	29.6	21.0	
Total Mediterranean diet scores	7.0 (SD 2.0)	7.2 (SD 1.9)	6.9 (SD 2.0)	0.214

Values are the mean and SD. Bold values indicate statistical significance *p* ≤ 0.05. BMI = body mass index, EI = energy intake. ^a^ Subsample, *n* = 239. ^b^ Sum of the standardized z-score of dynamometry/weight and standing long jump. * Student’s *t*-tests (continuous variables) or chi-squared tests (categorical variables).

**Table 2:** Agreement with the recommendations based on each item of the Mediterranean Diet Adherence Screener (MEDAS-14 item score), by sex.

	Recommendation	Agreement with the Recommendation		No Agreement with the Recommendation		*p*-Value (X^2^)
		Men	Women	Men	Women	
1. Do you use olive oil as a main culinary fat?	Yes	92.1	90.1	7.9	9.9	0.530
2. How much olive oil do you consume in a given day (including oil used for frying, salads, out-of-house meals, etc.)?	≥4 tbsp	39.7	28.9	60.3	71.1	**0.037**
3. How many vegetable servings do you consume per day? (1 serving: 200 g (consider side dishes as half a serving))	≥2 (≥1 portion raw or as a salad)	33.1	40.5	66.9	59.5	0.164
4. How many fruit units (including natural fruit juices) do you consume per day?	≥3	37.8	26.2	62.2	73.8	**0.022**
5. How many servings of red meat, hamburger, or meat products (ham, sausage, etc.) do you consume per day? (1 serving: 100–150 g)	<1	47.6	61.4	52.4	38.6	**0.012**
6. How many servings of butter, margarine, or cream do you consume per day? (1 serving: 12 g)	<1	94.4	95.7	5.6	4.3	0.585
7. How many sweet or carbonated beverages do you drink per day?	<1	88.8	94.4	11.2	5.6	0.062
8. How much wine do you drink per week?	≥7 glasses	2.6	1.4	97.4	98.6	0.460
9. How many servings of legumes do you consume per week? (1 serving: 150 g)	≥3	38.9	32.2	61.1	67.7	0.213
10. How many servings of fish or shellfish do you consume per week? (1 serving 100–150 g of fish or 4–5 units or 200 g of shellfish)	≥3	29.6	32.3	70.4	67.7	0.596
11. How many times per week do you consume commercial sweets or pastries (not homemade), such as cakes, cookies, biscuits, or custard?	<3	46.8	47.2	53.2	52.8	0.948
12. How many servings of nuts (including peanuts) do you consume per week? (1 serving 30 g)	≥3	26.2	18.0	73.8	82.0	0.069
13. Do you preferentially consume chicken, turkey, or rabbit meat instead of veal, pork, hamburger, or sausage?	Yes	70.1	70.4	29.9	29.6	0.951
14. How many times per week do you consume vegetables, pasta, rice, or other dishes seasoned with sofrito (sauce made with tomato and onion, leek, or garlic and simmered with olive oil)?	≥2	68.5	62.1	31.5	37.9	0.224

Values are percentages. Bold values indicate statistical significance *p* ≤ 0.05. Tbsp = tablespoon.

**Table 3:** The ANCOVA models comparing means of energy intake, body composition, and fitness parameters by MESA categories.

	Low Adherence (*n* = 74)	Medium Adherence (*n* = 160)	High Adherence (*n* = 76)	*p*
**Model 0**				
Weight (Kg)	62.9 (SD12.1)	64.3 (SD11.9)	67.6 (SD11.4) ^c^	**0.047**
Height (cm)	166.3 (SD8.5)	167.7 (SD9.1)	168.6 (SD8.4)	0.282
BMI (Kg/m^2^)	22.6 (SD3.1)	22.7 (SD3.1)	23.8 (SD3.8)	**0.050**
Waist circumference (cm)	77.4 (SD9.4)	78.0 (SD8.7)	80.1 (SD9.1)	0.419
Waist circumference(cm)/height (cm) index	0.465 (SD0.04)	0.465 (SD0.04)	0.475 (SD0.05)	0.297
% Fat mass	28.9 (SD9.2)	28.9 (SD9.6)	28.9 (SD10.2)	0.999
Total fat mass (Kg) ^a,^*	17.9 (SD6.3)	18.1 (SD6.1)	18.2 (SD6.6)	0.417
Total lean mass (Kg) ^a,^*	42.2 (SD6.0)	42.3 (SD6.0)	45.2 (SD6.4 ^c^)	**<0.001**
20 m SRT (stages)	5.4 (SD2.4)	5.6 (SD2.5)	6.5 (SD2.9 ^c^)	**<0.001**
CRF (VO_2_ max estimate)	32.4 (SD9.0)	32.8 (SD10.3)	35.6 (SD10.5 ^c^)	**<0.001**
Muscular strength index ^b^	−0.223 (SD1.6)	−0.096 (SD1.6)	0.350 (SD1.9 ^c^)	**<0.001**
Standing long jump (cm) *	153.1 (SD43.8)	161.3 (SD41.9)	167.0 (SD47.2 ^c^)	**<0.001**
Dynamometry (kg) *	29.2 (SD9.5)	29.3 (SD9.4)	31.9 (SD8.7 ^c^)	**<0.001**
Physical activity (min/day)	216.6 (SD58.8)	220.18 (SD81.25)	232.48 (SD64.30)	0.712
EI (Kcal)	2570.7 (SD967.7)	2883.9 (SD2301.0)	2955.2 (SD1594.6)	0.404
Carbohydrate (% EI)	42.9 (SD6.7)	42.6 (SD6.9)	43.8 (SD7.0)	0.428
Protein (% EI)	16.2 (SD2.7)	17.6 (SD3.4)	18.0 (SD3.4 ^c^)	**0.002**
Fat (% EI)	39.5 (SD5.5)	38.3 (SD6.1)	36.7 (SD6.3 ^c^)	**0.020**
**Model 1**				
Weight (Kg)	65.0 (SD11.3)	66.3 (SD12.0)	68.0 (SD11.4)	0.258
Height (cm)	168.8 (SD8.2)	169.8 (SD9.0)	169.6 (SD8.4)	0.596
BMI (Kg/m^2^)	22.6 (SD3.0)	22.8 (SD3.2)	23.4 (SD3.7)	0.155
Waist circumference (cm)	78.7 (SD9.0)	79.2 (SD8.8)	80.2 (SD9.1)	0.419
Waist circumference(cm)/height (cm) index	0.466 (SD0.04)	0.466 (SD0.04)	0.474 (SD0.05)	0.525
% Fat mass	23.9 (SD9.9)	23.9 (SD9.6)	24.0 (SD10.1)	0.997
Total fat mass (Kg) ^a,^*	16.0 (SD7.4)	16.1 (SD7.2)	17.1 (SD7.6)	0.725
Total lean mass (Kg) ^a,^*	42.5 (SD6.0)	42.7 (SD6.0)	43.1 (SD6.4)	0.595
20 m SRT (stages)	5.7 (SD2.4)	6.1 (SD2.6)	6.6 (SD2.6 ^c^)	**0.021**
CRF (VO_2_ max estimate)	33.4 (SD8.8)	34.8 (SD10.3)	36.5 (SD10.5 ^c^)	**0.017**
Muscular strength index ^b^	0.066 (SD1.6)	0.173 (SD1.6)	0.328 (SD1.7)	0.181
Standing long jump (cm) *	158.6 (SD43.8)	165.3 (SD41.9)	166.7 (SD47.2)	0.366
Dynamometry (kg) *	30.6 (SD9.0)	30.1 (SD9.4)	31.9 (SD8.6 ^d^)	**0.005**
Physical activity (min/day)	198.1 (SD60.9)	206.0 (SD84.2)	242.5 (SD73.6)	0.154
EI (Kcal)	2576.7 (SD964.5)	2889.7 (SD2340.0)	3013.1 (SD1594.6)	0.419
Carbohydrate (% EI)	42.9 (SD6.8)	42.7 (SD7.0)	43.8 (SD7.0)	0.566
Protein (% EI)	16.0 (SD2.7)	17.5 (SD3.4)	18.1 (SD3.4 ^c^)	**0.002**
Fat (% EI)	39.5 (SD5.6)	38.2 (SD6.2)	36.7 (SD6.3 ^c^)	**0.023**

Bold values indicate statistical significance *p* ≤ 0.05. Abbreviations: BMI = body mass index; EI = energy intake. ^a^ Subsample, *n* = 202. ^b^ Sum of the standardized z-score of dynamometry/weight and standing long jump, ^c^ statistical significance (*p* < 0.05) in pairwise mean comparisons using Bonferroni post-hoc test: ^c^ low < high; ^d^ medium < high *adjusted for height. Model 0: crude data; Model 1: adjusted for sex, age, smoking habits, alcohol habits, and type of housing.

**Table 4:** Logistic regression model predicting high adherence to the MD based on each item of the MD questionnaire.

	Reference (1)	OR (95% CI)	*p*-Value
*Questions from the MEDAS-14*			
1. Do you use olive oil as a main culinary fat?	Yes	10.6 (1.4–19.8)	**0.021**
2. How much olive oil do you consume in a given day (including oil used for frying, salads, out-of-house meals, etc.)?	≥4 tbsp	2.8 (1.6–4.7)	**<0.001**
3. How many vegetable servings do you consume per day? (1 serving: 200 g (consider side dishes as half a serving)])	≥2 (≥1 portion raw or as a salad)	20.1 (10.1–30.1)	**<0.001**
4. How many fruit units (including natural fruit juices) do you consume per day?	≥3	8.8 (4.9–15.7)	**<0.001**
5. How many servings of red meat, hamburger, or meat products (ham, sausage, etc.) do you consume per day? (1 serving: 100–150 g)	<1	2.8 (1.5–4.9)	**<0.001**
6. How many servings of butter, margarine, or cream do you consume per day? (1 serving: 12 g)	<1	2.1 (0.4–9.8)	0.313
7. How many sweet or carbonated beverages do you drink per day?	<1	6.6 (0.8–50.3)	0.068
8. How much wine do you drink per week?	≥7 glasses	6.4 (1.1–35.9)	**0.034**
9. How many servings of legumes do you consume per week? (1 serving: 150 g)	≥3	3.0 (1.7–5.1)	**<0.001**
10. How many servings of fish or shellfish do you consume per week? (1 serving 100–150 g of fish or 4–5 units or 200 g of shellfish)	≥3	4.8 (2.7–8.3)	**<0.001**
11. How many times per week do you consume commercial sweets or pastries (not homemade), such as cakes, cookies, biscuits, or custard?	<3	10.1 (5.1–19.7)	**<0.001**
12. How many servings of nuts (including peanuts) do you consume per week? (1 serving 30 g)	≥3	5.3 (2.9–9.4)	**<0.001**
13. Do you preferentially consume chicken, turkey, or rabbit meat instead of veal, pork, hamburger, or sausage?	Yes	3.0 (1.5–6.0)	**0.002**
14. How many times per week do you consume vegetables, pasta, rice, or other dishes seasoned with sofrito (sauce made with tomato and onion, leek, or garlic and simmered with olive oil)?	≥2	3.2 (1.7–6.0)	**<0.001**

Values are proportions. Bold values indicate statistical significance *p* ≤ 0.05; Tbsp = tablespoon.
